# Outpatient parenteral antimicrobial therapy in pediatrics: the role of antimicrobial stewardship

**DOI:** 10.1017/ash.2024.405

**Published:** 2024-11-13

**Authors:** Daniel J. Trisno, Lauren Puckett, Alexandria Jensen, Hayden T. Schwenk

**Affiliations:** 1 Department of Pharmacy, Lucile Packard Children’s Hospital Stanford, Palo Alto, CA, USA; 2 Quantitative Sciences Unit, Stanford School of Medicine, Stanford, CA, USA; 3 Department of Pediatrics, Division of Pediatric Infectious Diseases, Stanford School of Medicine, Stanford, CA, USA

## Abstract

This study characterized outpatient parenteral antimicrobial therapy (OPAT) orders and associated antimicrobial stewardship program (ASP) pharmacist recommendations made in a freestanding children’s hospital. Recommendations occurred in over 50% of orders, indicating an opportunity for the review of OPAT by ASP pharmacists.

## Introduction

Although the use of outpatient parenteral antimicrobial therapy (OPAT) may be beneficial in certain scenarios, OPAT coordination is a complex, multidisciplinary process that requires communication between healthcare providers to optimize success and minimize risks.^1,2^ The Infectious Diseases Society of America (IDSA) recommends that all patients have their OPAT reviewed by an infectious diseases (ID) expert prior to initiation; however, there are currently no recommendations for routine review by antimicrobial stewardship programs (ASPs).^3^

A growing number of US and international hospitals have implemented ASP review of OPAT.^4,5^ Unique features of OPAT ASP review include consideration of medication administration and stability.^1^ In adult patients, ASP review of OPAT has been shown to lead to more optimal antimicrobial therapy.^4^ Although similar opportunities for OPAT optimization have been reported in the pediatric literature, there is a lack of recent reports on pediatric OPAT utilization.^5–7^

The primary aim of this study was to provide a contemporary description of the characteristics of pediatric OPAT. We also sought to identify the frequency of ASP recommendations and explore potential associative variables with prescriptions requiring ASP intervention.

## Methods

An 18-month single-center, retrospective chart review was performed at a 461 bed quaternary children’s hospital of OPAT orders since the program began in March 2021. We included OPAT orders of at least 2 days duration dispensed through the hospital’s affiliated home infusion pharmacy, Children’s Home Pharmacy (CHP).

At our hospital, an ASP pharmacist performs prospective audit and feedback (PAF) for inpatient intravenous (IV) antimicrobial orders. A second, outpatient ASP pharmacist performs PAF on discharge enteral antimicrobial orders and OPAT prescriptions dispensed from CHP, who notify ASP of prescribed OPAT orders. Most patients are managed by their primary team after discharge and ID consultation is not required before OPAT prescribing. OPAT audit data elements include patient demographics, prescription details, indication, prescribing medical service, whether OPAT is recommended per ID, culture results, concomitant IV medications, whether an ASP recommendation is made, and whether recommendations are followed. Patients are also reviewed for associated inpatient PAF (ie, PAF for the same antimicrobial and indication within the same medical encounter).

For this study, ASP recommendations were categorized into care coordination, administration, stop OPAT, modify order, or change antimicrobial (Table S1). The number of recommendations made, as well as the proportion of recommendations accepted, were aggregated.

To determine if specific variables were associated with odds of ASP intervention, data pertaining to prescribing service, indication, and antimicrobial type were collapsed into higher-level categories for analysis. Prescribing service was categorized into acute care, immunocompromised services, and other. Indication was grouped as bloodstream infections, prophylaxis, and other. Antimicrobials were stratified by type, with antibiotics further stratified into National Healthcare Safety Network (NHSN) pediatric antibiotic spectrum groupings.^8^

Patient demographics and descriptive audit characteristics were summarized using medians and interquartile ranges (IQRs). A multivariate logistic regression was fit to determine if any of the collected data elements were associated with odds of ASP pharmacy intervention. Statistical significance was determined at the standard *P* < 0.05 level. All analyses were conducted using R (version 4.3.0; Vienna, Austria).

## Results

A total of 104 OPAT orders were reviewed by the ASP during the study period, 93 of which met inclusion criteria. Of the 93 included orders, there were 66 unique patients over 84 encounters. Table 1 depicts the characteristics of OPAT orders included in this study. Excluding antimicrobials prescribed for prophylaxis, the median duration of OPAT courses was 10 days (IQR: 5.0–16.5) and most orders were associated with a positive blood culture (72%).

**Table 1.** Characteristics of OPAT order

Characteristics	Number (N = 93)
	N (%)
Age (years)	
0–<2	11 (11.8)
2–12	32 (34.4)
>12	50 (53.8)
Service	
Acute care	46 (49.4)
Gastroenterology	20 (21.5)
General pediatrics	11 (11.8)
Cardiology	6 (6.5)
Pulmonology	5 (5.4)
Liver transplant	3 (3.2)
Nephrology	1 (1.1)
Immunocompromised services	40 (43)
Hematology/oncology	33 (35.5)
Stem cell transplant	7 (7.5)
Other	7 (7.5)
Surgery	4 (4.3)
Intensive care unit	3 (3.2)
Infectious problem	
Bloodstream infections	39 (41.9)
Bacteremia	21 (22.6)
CLABSI	12 (12.9)
Fungemia	6 (6.5)
Prophylaxis	17 (18.3)
Other	37 (39.8)
Central nervous system	10 (10.8)
Respiratory	8 (8.6)
Endocarditis	4 (4.3)
Intra-abdominal	4 (4.3)
Viral	4 (4.3)
UTI	3 (3.2)
SSTI	2 (2.2)
Osteomyelitis	2 (2.2)
Positive culture results	55 (59.1)
Concomitant parenteral medications	34 (36.6)
ID consult	51 (54.8)
Inpatient PAF conducted	64 (68.8)

CLABSI, central line-associated bloodstream infection; UTI, urinary tract infection; SSTI, skin and soft tissue infection.

Antibiotics were the most frequently prescribed type of antimicrobial (71%), with a relatively even distribution among the NHSN pediatric antibiotic spectrum groups (Figure S1). Ceftriaxone (28%) and vancomycin (14%) were the most frequently prescribed antibiotics. Sixty-three (68%) OPAT orders had an ASP recommendation and 49 (77%) were accepted (Figure S2). Of the recommendations made to stop OPAT, four were for a change from IV to PO, and none were accepted.

**Figure 1.**
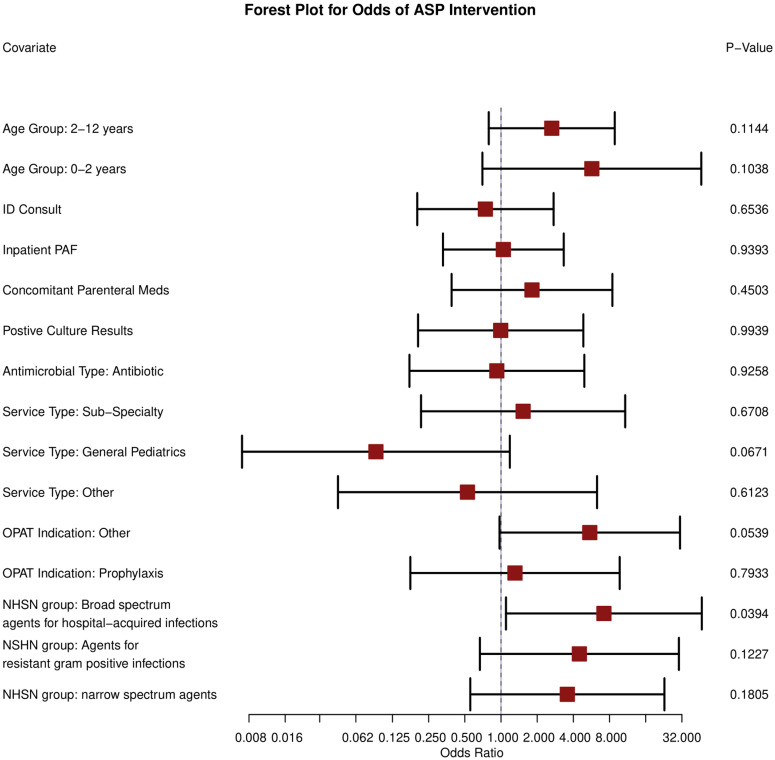
Forest plot for predictors of ASP intervention. Reference categories are not presented, including age group: >12 years, service type: immunocompromised, OPAT indication: BSI, and NSHN group: broad-spectrum community.

Within the multivariate logistic regression, only one variable—the NHSN group of broad-spectrum agents for hospital-acquired infections (ie, ertapenem, piperacillin-tazobactam, ceftazidime, and cefepime)—was found to be significantly associated with increased odds of an ASP recommendation (OR; 7.18; 95% CI: 1.10–46.87; *P* = 0.04) (Figure 1). Care coordination was the most common ASP recommendation observed.

## Discussion

This study describes the role of ASP review of pediatric OPAT orders at our institution and highlights the opportunity for other ASP programs to implement a similar practice to optimize OPAT orders. Broad-spectrum gram-negative agents were significantly associated with a higher odds of intervention by the ASP when used for OPAT, even when inpatient use of these medications had been previously reviewed by the ASP. This may be due to specific intervention opportunities available for agents within this category. For example, cefepime and piperacillin-tazobactam were frequently changed from intermittent to prolonged infusion to optimize pharmacokinetics and promote ease of administration. These findings underscore the importance of discharge antimicrobial stewardship, even at institutions with robust inpatient stewardship processes.

Compared to earlier reports of pediatric OPAT utilization, we found little use of OPAT for either osteoarticular infections or pneumonia.^6,7^ This is likely driven by contemporary literature supporting the use of enteral agents for these indications.^9^ Importantly, we found that prophylaxis was the second most common indication for OPAT. Orders for prophylaxis were per protocol as supportive care for patients with acute lymphoblastic leukemia and lymphoma (eg, caspofungin antifungal prophylaxis). There was no statistically significant difference in the rate of ASP intervention for these OPAT prescriptions. ASP review of all prescriptions may be beneficial, particularly at centers with large numbers of immunocompromised children.

Only 55% of OPAT orders in this study were associated with an ID consultation, despite IDSA guidelines recommending one for all OPAT courses.^3^ Though ID consultation has been noted to improve outcomes with OPAT orders, in our study, it did not affect whether OPAT recommendations were made by the ASP at the time of discharge.^10^ Similarly, prior inpatient PAF of OPAT orders was not associated with lower rates of ASP recommendation, highlighting unique opportunities for ASP review of discharge OPAT prescriptions.

Our study had a higher rate of ASP recommendations (68%) than previously published.^5^ Most ASP recommendations were for care coordination, a recommendation category not captured in prior studies. Although these recommendations were primarily for additional outpatient monitoring, 20% were to discontinue unnecessary lab monitoring. ASP review of OPAT orders may be the only opportunity to ensure appropriate toxicity monitoring plans for the outpatient setting.

This was a single-center study, which limits the generalizability of our findings to other institutions. Only OPAT orders through CHP (90% of total orders) were captured in this study. A formal power calculation was not performed, and the study sample size was small. As a result, some confidence intervals within the multivariate logistic regression analysis were markedly wide, making it difficult to draw definitive conclusions.

Our study demonstrates that ASP review of discharge orders can potentially improve the quality of OPAT prescribing, even for patients followed by ID, and should be considered by pediatric ASPs. Prospective studies with larger sample sizes and that assess clinical outcomes are needed to better characterize the impact of this intervention in pediatric patients.

## Supporting information

Trisno et al. supplementary material 1 Trisno et al. supplementary material

Trisno et al. supplementary material 2 Trisno et al. supplementary material

Trisno et al. supplementary material 3 Trisno et al. supplementary material

## Supplementary material

The supplementary material for this article can be found at https://doi.org/10.1017/ash.2024.405.
