# Supplementary figures and images for: Outpatient parenteral antimicrobial therapy in pediatrics: the role of antimicrobial stewardship
Source: Antimicrob Steward Healthc Epidemiol. 2024 Nov 13;4(1):e203. doi: 10.1017/ash.2024.405 (PMC11574587; doi:10.1017/ash.2024.405)

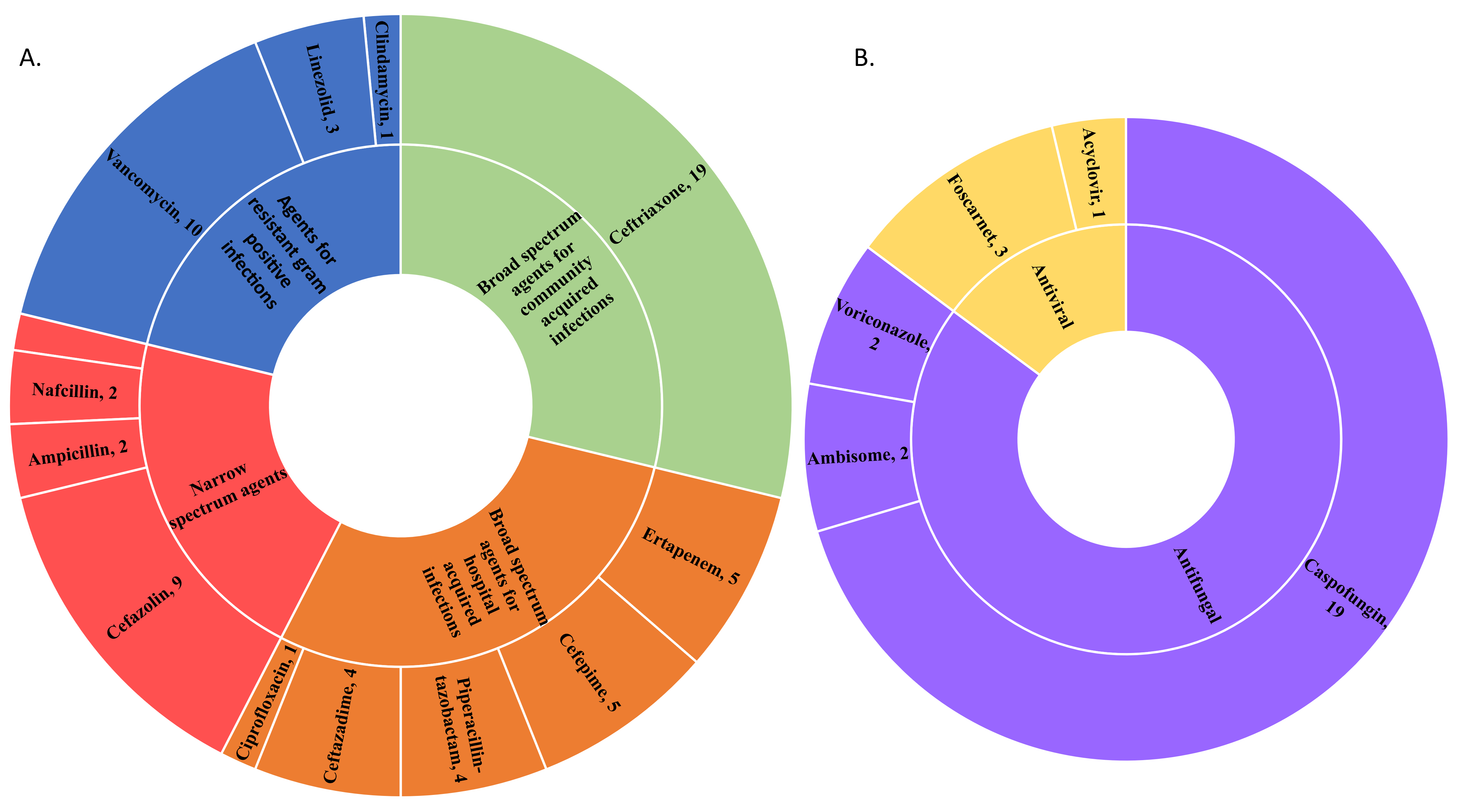

Supplement: Trisno et al. supplementary material 1 — Trisno et al. supplementary material [file S2732494X24004054sup001.tif]

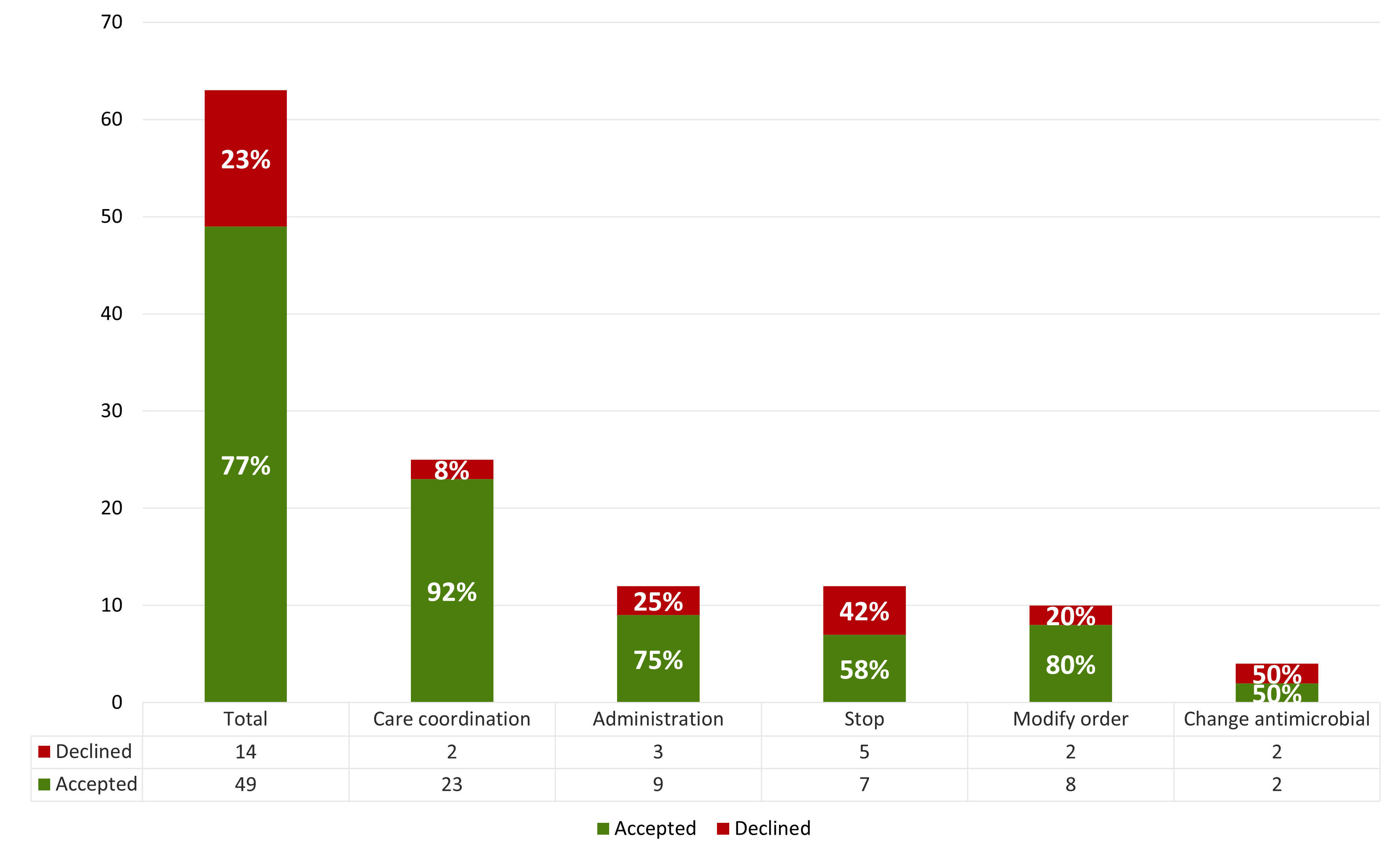

Supplement: Trisno et al. supplementary material 2 — Trisno et al. supplementary material [file S2732494X24004054sup002.tif]
