# Supplementary material for: Outpatient parenteral antimicrobial therapy in pediatrics: the role of antimicrobial stewardship
Source: Antimicrob Steward Healthc Epidemiol. 2024 Nov 13;4(1):e203. doi: 10.1017/ash.2024.405 (PMC11574587; doi:10.1017/ash.2024.405)

**Supplementary Table 1. Recommendations made under each category**

| Recommendation category | Types of recommendations |
| --- | --- |
| Care coordination | Monitoring (e.g., Increase/decreasing monitoring frequency, discontinue ordered labs) |
|  | Retiming of antimicrobials (e.g. Timing inpatient doses to accommodate home administration) |
|  | Test dose coordination |
| Stop OPAT | Discontinuation |
|  | Switch to enteral alternative |
| Modify order | Adjust frequency |
|  | Adjust duration (e.g. Shorten duration) |
|  | Infusion type (e.g. Recommend extended infusion) |
| Administration | Compatibility considerations |
| Change antimicrobial | Narrow agent |

**Supplementary Figure 1. Sunburst plot of antimicrobial breakdown. A) NHSN pediatric antibiotic group breakdown, B) Antifungal and antiviral breakdown**


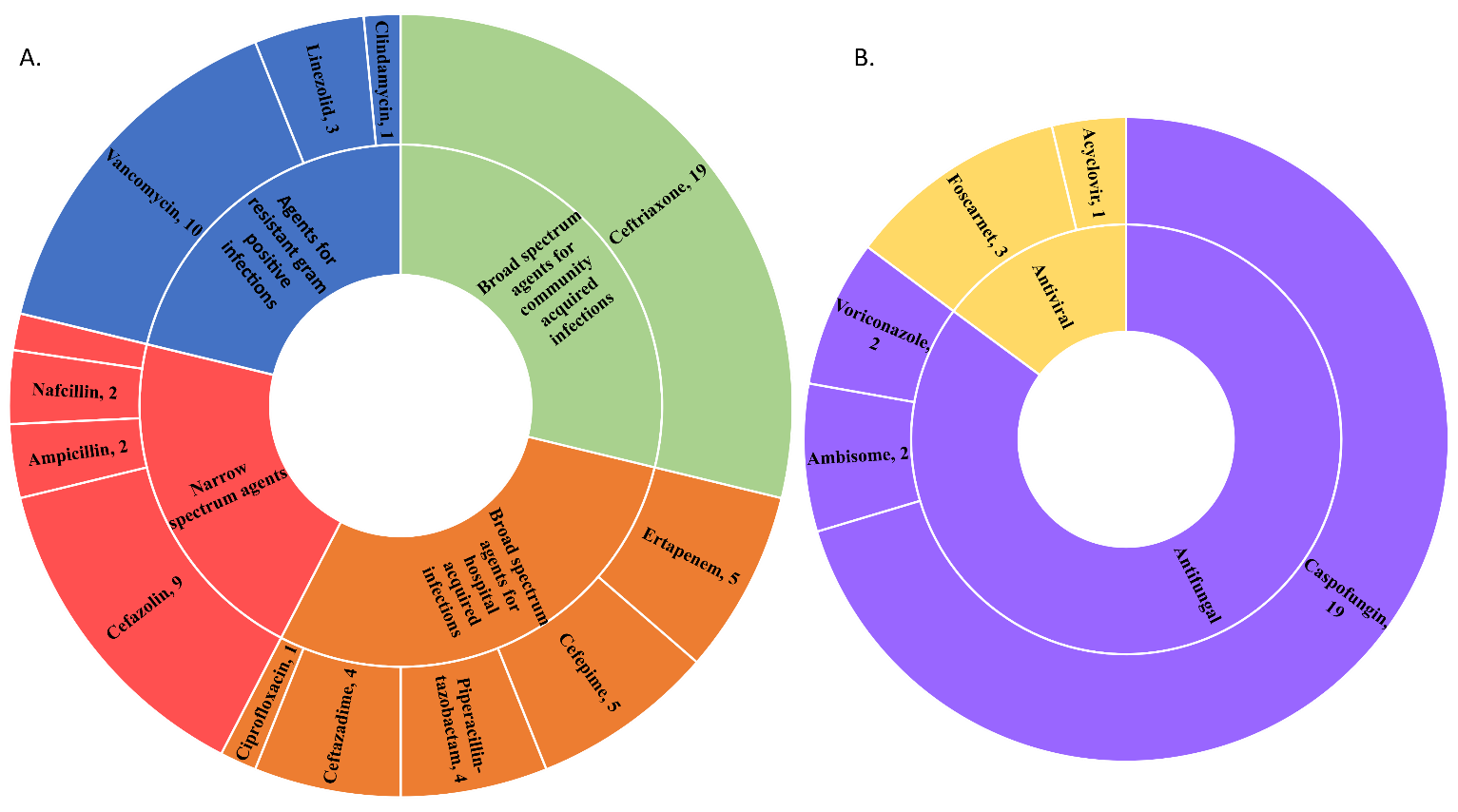


**Supplementary Figure 2. Breakdown of ASP recommendations with percent of recommendations accepted**


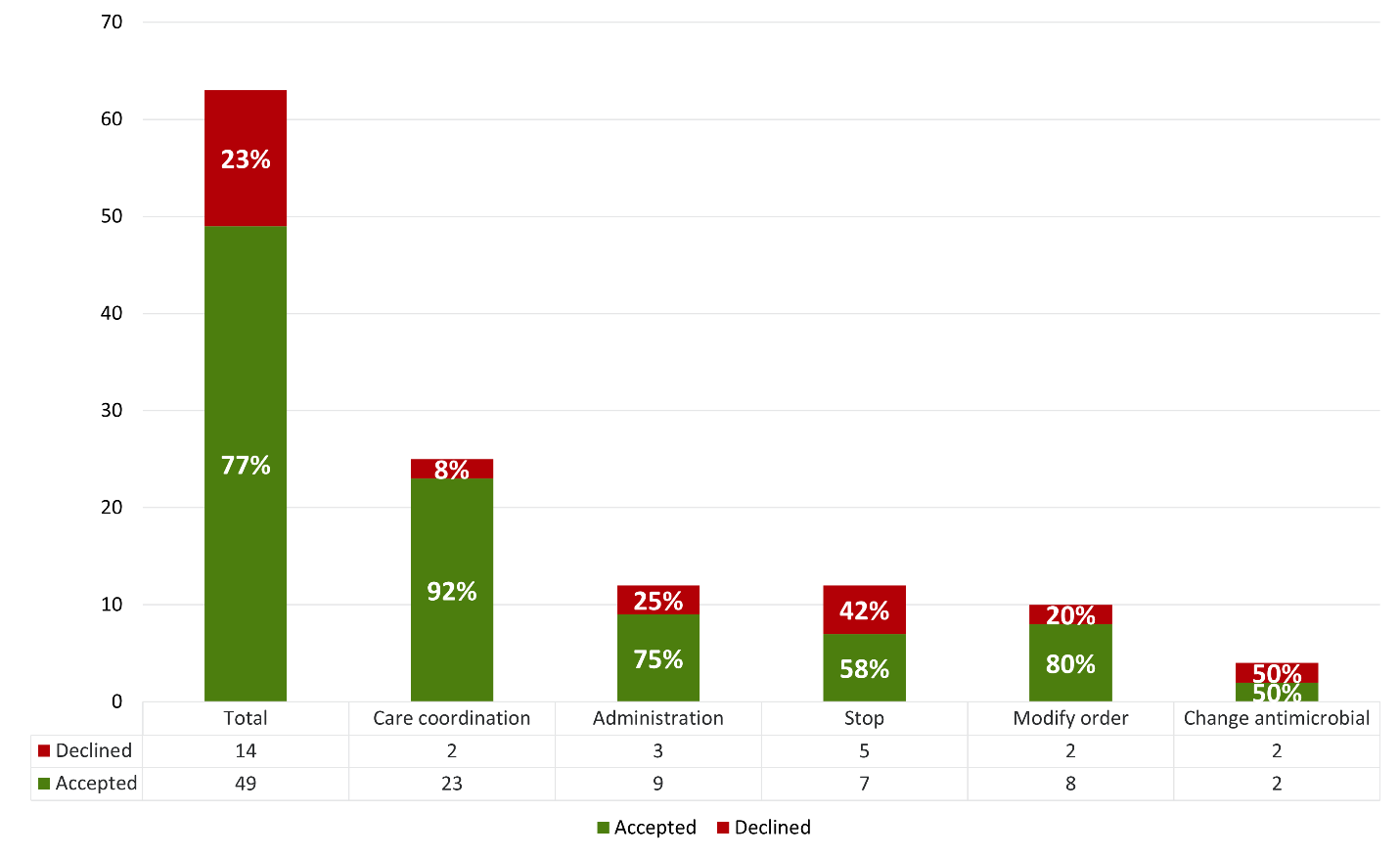

Supplement: Trisno et al. supplementary material 3 — Trisno et al. supplementary material [file S2732494X24004054sup003.docx]
